# The secretome from bovine mammosphere-derived cells (MDC) promotes angiogenesis, epithelial cell migration, and contains factors associated with defense and immunity

**DOI:** 10.1038/s41598-018-23770-z

**Published:** 2018-03-29

**Authors:** Melissa M. Ledet, Amy K. Vasquez, Gat Rauner, Allison A. Bichoupan, Paolo Moroni, Daryl V. Nydam, Gerlinde R. Van de Walle

**Affiliations:** 1 Baker Institute for Animal Health, College of Veterinary Medicine, Cornell University, Ithaca, NY United States; 2 Department of Population Medicine and Diagnostic Sciences, College of Veterinary Medicine, Cornell University, Ithaca, NY United States

## Abstract

Treatment of bovine mastitis with intramammary antibiotics is common, yet several concerns exist including failed efficacy for individual hosts or pathogens and the inability of approved drugs to revert mastitis-induced tissue damage to healthy tissue capable of returning to full milk production. These issues, in addition to aspects of public health such as accidental antibiotic residues in saleable milk and the potential for antimicrobial resistance, support the need to find alternative therapies for this costly disease. This study shows that the secretome, or collective factors, produced by mammosphere-derived cells (MDC) promotes angiogenesis, epithelial cell migration, and contains proteins associated with immunity and defense; all of which are necessary for healing damaged mammary gland tissue. Furthermore, we found that the MDC secretome remains effective after freezing and thawing, enhancing its therapeutic potential. Our results provide a foundation for further characterization of the individual secreted factors and the rationale for using the MDC secretome as a complementary treatment for bovine mastitis.

## Introduction

Mastitis is defined as inflammation of the mammary gland, and the main etiological contributors in dairy animals are bacterial in origin. Clinical and subclinical mastitis, highly prevalent diseases in the dairy industry, have considerable economic impacts with contributions to milk production losses, milk quality concerns, labor costs, and reproductive deficiencies^1,2^; each case, when occurring in early lactation, is estimated to cost approximately $444^3^. Gram-negative coliform bacteria acquired from the environment, such as *Klebsiella (K*.) *pneumoniae*, frequently cause an acute inflammatory response with severe clinical signs, while Gram-positive contagious pathogens such as *Staphylococcus (S*.*) aureus* may cause persistent, subclinical and chronic infections.

In addition to bacterial burden, damage caused to the mammary gland during mastitis reduces the number and activity of epithelial cells through disruption of alveolar cell integrity, sloughing of cells and induced apoptosis. This destruction will lead to a build-up of milk constituents in the secretory epithelium resulting in a breakdown of the basement membrane due to stromal thickening. This results in a decreased percentage of tissue areas occupied by alveolar epithelium and lumina and an increased percentage of interalveolar stromal areas^4^. The decrease in secretory epithelium consequently contributes to approximately 70% of the total cost of mastitis^4^.

The most common use for antibiotics on dairy farms is for the prevention of intramammary infections (IMI) and treatment of mastitis^5^. A survey performed by the USDA in 2014 showed that 21.7% of cows experiencing clinical mastitis are treated with antibiotics, and 96.9% of dairy facilities use antibiotics to treat clinical mastitis cases^6^. Several advantages of antibiotic use for the treatment of mastitis have been reported and include faster clearance of bacteria, increased survival rate of cows, and reduction in losses of milk production^7^. However, the treatment of mastitis caused by coliform organisms such as *K*. *pneumoniae* with antibiotics alone is difficult because it is often characterized by massive inflammation and widespread udder tissue necrosis, primarily caused by the bacterial toxin lipopolysaccharide (LPS)^8,9^. As such, an important limitation of antibiotics is their inability to fully revert the mastitis-induced epithelial structural damage in the udder to healthy pre-infection tissue capable of full milk production. Finally, use of antibiotics is directly related to the risk of residues in bulk tank milk, and the possible relationship of antimicrobial use to the emergence of resistance indicate reasons for public concern^10^.

Recent studies have shown that the cellular secretome, comprised of all secreted factors, plays an important role in various physiological processes, including cellular cross-talk and tissue regeneration^11,12^. The secretome of mesenchymal stem cells (MSC), a type of adult multipotent stem cells, is especially being studied in great depth due to its potential as a novel, stem cell-free, therapeutic strategy^13,14^. The MSC secretome contributes to healing processes by participating in the inflammatory, proliferative and remodeling phases of tissue repair, and can enhance bacterial clearance via the production of antimicrobial peptides (AMP)^15,16^. Based on these reported secretome properties and the need for alternative and/or adjunct therapies for mastitis, we decided to characterize the secretome of primary cells from the bovine mammary gland with an emphasis on potential regenerative and antimicrobial properties.

## Results

### Isolation of adherent fraction-derived cells (AFDC) and mammosphere-derived cells (MDC) from the bovine mammary gland yields two distinct populations

In order to study the secretome of bovine mammary cells, we isolated cells from fresh mammary tissue and cultured two different populations (Fig. S1). After enzymatic digestion, single cells were plated on a tissue culture dish for one hour. The population of adherent cells was collected and propagated as adherent fraction-derived cells (AFDC). The population of suspended cells was collected separately and propagated as mammospheres, a technique known to enrich for mammary stem/progenitor cells^17,18^. After an eleven-day selection period, these mammospheres were cultured on regular tissue culture plates and propagated as mammosphere-derived cells (MDC). AFDC and MDC were morphologically distinct from each other, with AFDC being morphologically heterogenic with some of the cells being epithelial-like while others displayed an elongated spindle-shaped morphology, and MDC being primarily epithelial-like with polygonal shapes that grew in discrete patches (Fig. S1).

**Figure 1:**
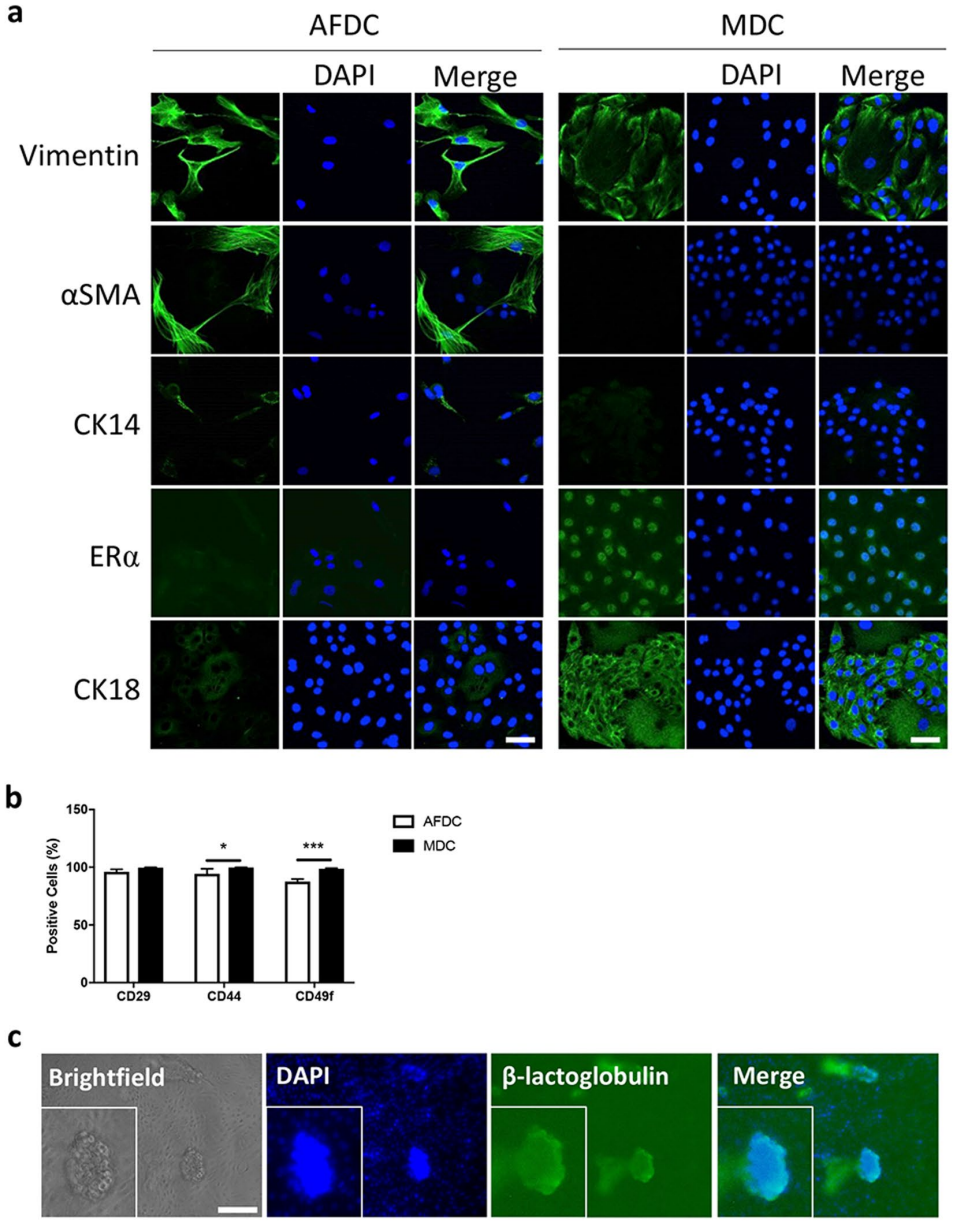
AFDC and MDC are distinct populations. (**a**) Immunofluorescence staining of AFDC and MDC, demonstrating expression of the mammary cell markers vimentin (expressed by both populations), αSMA, CK14 (expressed only in AFDC), ERα and CK18 (expressed only in MDC). Bars = 50 µm. (**b**) Flow cytometry was performed with three mammary cell surface markers: CD29, CD44 and CD49f. The percentage of positive cells was quantified compared to a relevant isotype control. (**c**) Immunofluorescence of acinar structures expressing β-lactoglobulin (green). Inset: enlargement of representative acinar structure. Nuclei were stained with DAPI (blue). Bar = 100 µm; *p < 0.05, ***p < 0.001.

We further characterized these two cell populations based on their expression of five mammary gland markers by immunofluorescence (IF). Vimentin, CK14 and alpha smooth muscle actin (αSMA) are markers of myoepithelial cells^19–22^. Estrogen receptor alpha (ERα) and CK18 are markers of luminal epithelial cells^21,23^. Both cell populations expressed vimentin, while only AFDC expressed αSMA and CK14, and only MDC expressed ERα and CK18 (Fig. 1a). The IF was complemented with flow cytometry analysis to investigate the expression of three cell surface markers commonly used to immunophenotype mammary cells^21,24,25^. We found that CD29 was highly expressed on both cell types, but MDC expressed statistically more CD44 and CD49f compared to AFDC (Fig. 1b). Collectively, these results show that we isolated two distinct bovine cell populations with different morphology and, at least in part, a different protein expression pattern. Since mammospheres are known to be enriched in cells with stem/progenitor characteristics, as described above, we decided to determine the potential of the bovine MDC population to differentiate into milk-producing cells. To this end, cells were cultured on Matrigel in the presence of prolactin. Acinar structures could readily be detected after 14 days in culture (Fig. 1c) and these structures were positive for β-lactoglobulin using IF. These results show that the primary bovine MDC cultures, obtained through propagation as non-adherent mammospheres, indeed retain their potential to differentiate into milk-secreting luminal epithelial cells *in vitro*.

### Characterization of the secretomes from MDC and AFDC using mass spectrometry reveals secreted factors with antimicrobial and regenerative properties

Liquid chromatography-mass spectrometry was used to globally characterize the secretome, collected as conditioned medium (CM), from both MDC and AFDC. To this end, MDC and AFDC CM was submitted for proteomic analysis and yielded 347 and 537 matched proteins from peptide sequences in MDC and AFDC CM, respectively. Table 1 lists the identified proteins related to defense and immunity and regeneration, such as angiogenesis and cell migration. Selected proteins from each category were then quantified using Western blot analyses or commercially available enzyme-linked immunosorbent assays (ELISAs).

**Table 1:** Proteins identified in the conditioned medium (CM) of bovine mammosphere-derived cells (MDC) and adherent fraction-derived cells (AFDC) by mass spectrometry.

MDC	AFDC
***Proteins having a role in defense and immunity***	
Alpha-2-macroglobulin	Alpha-2-macroglobulin
Cathepsin	Cathepsin
Alpha-1B-glycoprotein	Alpha-1B-glycoprotein
Pantetheinase	Pantetheinase
Beta-2-microglobulin	Beta-2-microglobulin
Protein S100-A11	Protein S100-A11
Mannose-binding protein C	Mannose-binding protein C
Lactoferrin	Lactoferrin
Peptidoglycan recognition protein	Collectin-43
Cathelicidin-7	Cathelicidin-1
Contactin-1	Contactin-1
Collectin-11	Collectin-11
Zinc-alpha-2-glycoprotein	Zinc-alpha-2-glycoprotein
	Phospholipid transfer protein
	Interleukin-6 receptor subunit beta
	Hepatitis A virus cellular receptor 1
***Proteins having a role in angiogenesis***	
Angiopoitin-related protein 3 precursor	Angiopoitin-related protein 3 precursor
Vascular endothelial growth factor A isoform 4 precusor	
***Proteins having a role in cell migration***	
Hepatocyte growth factor activator preproprotein	Hepatocyte growth factor activator preproprotein
Hepatocyte growth factor-like protein isoform X1	Hepatocyte growth factor-like protein isoform X1
Hepatocyte growth factor receptor precursor	Insulin-like growth factor binding protein 4 precursor
Insulin-like growth factor binding protein 2 precursor	Insulin-like growth factor binding protein 2 precursor
Insulin-like growth factor binding protein 6 precursor	Insulin-like growth factor binding protein 6 precursor
Insulin like growth factor isoform X1	Insulin like growth factor isoform X1
Insulin-like growth factor binding protein complex acid labile subunit precursor	Insulin-like growth factor binding protein complex acid labile subunit precursor
Transforming growth factor beta induced protein ig-h3 precursor	Transforming growth factor beta induced protein ig-h3 precursor
Fibroblast growth factor binding protein 1 precursor	Transforming growth factor beta-2 isoform X1

**Figure 2:**
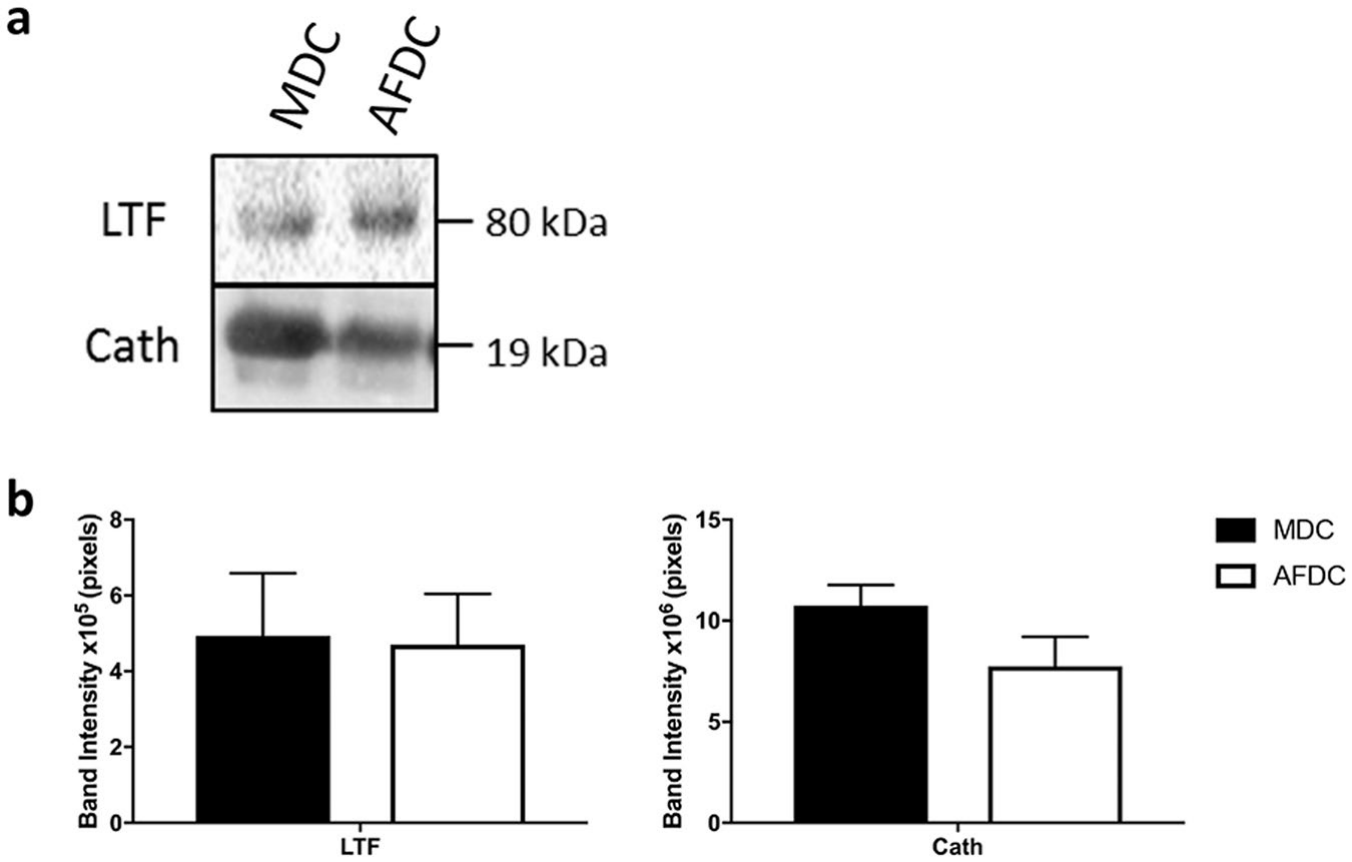
MDC and AFDC secrete antimicrobial peptides (AMP). (**a**) Representative images of protein lysates run on polyacrylamide gels, transferred to PVDF membranes, and probed with anti-AMP antibodies. 40 μg of each sample was loaded per lane. Lane 1 = MDC CM; Lane 2 = AFDC CM. (**b**) Band densities of two AMPs in MDC and AFDC CM as detected by western blot. *p < 0.05.

For the defense and immunity category we selected lactoferrin (LTF) and cathelicidin (Cath), both of which have previously been described to act as antimicrobial peptides (AMP)^26–29^. Since commercially available ELISA were unable to detect these proteins within the given detection limit (data not shown), Western blot analyses were used. Both LTF and Cath were detected in the CM of both bovine cell populations (Fig. 2). Vascular endothelial growth factor a (VEGFa) and angiopoetin 1 (Ang-1) were selected from the angiogenesis category. Quantification by ELISA showed that the AFDC CM contained higher levels of VEGFa, whereas the MDC CM contained higher levels of Ang-1 (Table 2). Lastly, Transforming Growth Factor Beta (TGFβ), Insulin-like Growth Factor-1 (IGF-1), and Hepatocyte Growth Factor (HGF) were selected from the migration category. Higher levels of TGFβ were detected by ELISA in MDC CM, whereas IGF-1 and HGF were equally expressed in both MDC and AFDC CM (Table 2).

**Table 2:** Quantification of secreted factors in bovine mammosphere-derived cells (MDC) and adherent fraction-derived cells (AFDC) using sandwich ELISA. Values represent the average ± standard deviation of three biological replicates.

Protein	Abbreviation	MaSC	BMF	P-value
**Angiogenesis**				
Vascular endothelial growth factor a	VEGFa	312 ± 15 pg/ml	568 ± 47 pg/ml	0.004
Angiopoitin 1	Ang-1	6150 ± 685 pg/ml	3629 ± 821 pg/ml	0.017
**Migration**				
Transforming growth factor beta	TGFβ	1916 ± 611 pg/ml	460 ± 343 pg/ml	0.02
Insulin-like growth factor 1	IGF-1	82 ± 16 pg/ml	64 ± 2 pg/ml	0.156
Hepatocyte growth factor	HGF	57 ± 49 pg/ml	19 ± 17 pg/ml	0.309

^*^NA: not applicable.

Collectively, these results show that both MDC and AFDC secrete various factors with roles in immune defense and regeneration. Using a series of *in vitro* experiments, we subsequently determined whether these secreted factors also had functional consequences.

### MDC and AFDC CM protect epithelial cells from LPS-induced cell damage

**Figure 3:**
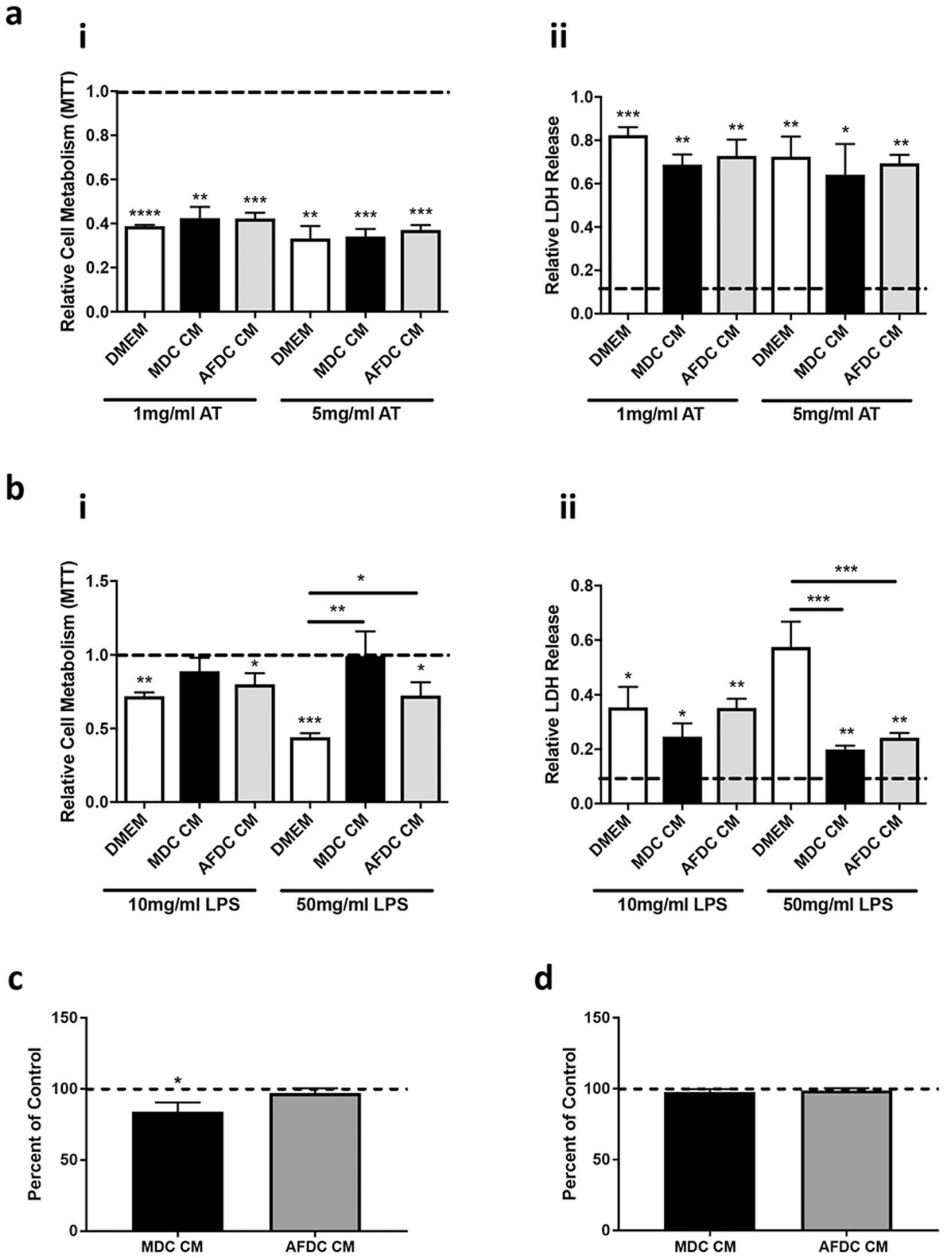
MDC and AFDC CM prevent epithelial cell damage from LPS but not alpha toxin. Madin-Darby Bovine Kidney Epithelial (MDBK) cells were treated with two different concentrations of alpha toxin (AT) (**a**) or lipopolysaccharides (LPS) (**b**) with or without MDC or AFDC CM. MTT (3-(4,5-dimethylthiazol-2-yl)-2,5-diphenyltetrazolium bromide; i) and LDH (lactate dehydrogenase) release assays (ii) were used to determine viability. (**c**) Inhibitory effect of 50% MDC CM and AFDC CM on *K*. *pneumoniae* as a percentage of the positive control (CM-free DMEM) indicated by the dashed line; growth was measured after an 8 h incubation (37 °C) as optical density at 600 nm in a 96-well plate (* indicates MDC being significantly different from control at *P* < 0.05). (**d**) Inhibitory effect was not present when growth was measured as a colony forming unit (CFU) count on LB agar after serial dilution and overnight incubation (16 h; 37 °C) of wells in (C). *p < 0.05, **p < 0.01, ***p < 0.001.

An important consequence of mastitis is epithelial cell damage caused by bacterial toxins. To determine if the secreted factors with roles in defense and immunity (Table 1) have a functional effect, we evaluated the effects of the MDC and AFDC CM on toxin-induced epithelial cell death. To this end, Madin-Darby Bovine Kidney Epithelial (MDBK) cells were treated with alpha-toxin (AT), a toxin produced by the Gram-positive *Staphylococcus* (*S*.*) aureus*, and lipopolysaccharide (LPS), a toxin produced by Gram-negative bacteria, such as *Klebsiella* (*K*.*) pneumoniae*, in the presence or absence of CM. As expected, both toxins (at concentrations of 10 and 50 mg/mL LPS and 1 and 5 mg/mL AT) induced a significant reduction in epithelial cell viability relative to untreated cells, as measured by a reduction in cell metabolism (3-(4,5-dimethylthiazol-2-yl)-2,5-diphenyltetrazolium bromide; MTT assay) and an increase in lactate dehydrogenase (LDH) release (Fig. 3a & b). No protective effect of the CM from both MDC and AFDC was observed in AT-treated cells (Fig. 3a). In contrast, a protective effect on cell viability was observed for the CM from either cell types on LPS-treated epithelial cells for cells treated with 50 mg/ml LPS (Fig. 3b). The combination of these findings, in addition to the observed presence of AMPs in the secretome, encouraged our group to consider the direct effects of CM on Gram-negative bacteria. To this end, we cultured the Gram-negative coliform mastitis pathogen *K*. *pneumoniae* in the presence or absence of MDC and AFDC CM. We measured bacterial growth as optical density (OD) and colony forming unit (CFU) counts, which we determined to have a correlation at log-phase growth of 0.95. Compared to bacteria grown in the absence of CM (control), a statistical reduction in OD was observed for bacteria grown in MDC, but not AFDC, CM, indicating an effect of the MDC CM on the growth of *K*. *pneumoniae* (Fig. 3c). However, as no differences in CFU counts were observed between bacteria grown in the presence of MDC CM and non-CM cultured bacteria (Fig. 3d), these effects are most likely bacteriostatic in nature, rather than bacteriocidal. As expected, no reduction in CFU was observed when bacteria were grown in AFDC CM (Fig. 3d).

### MDC CM stimulates angiogenesis not by proliferation, but by the promotion of bovine endothelial tube formation

**Figure 4:**
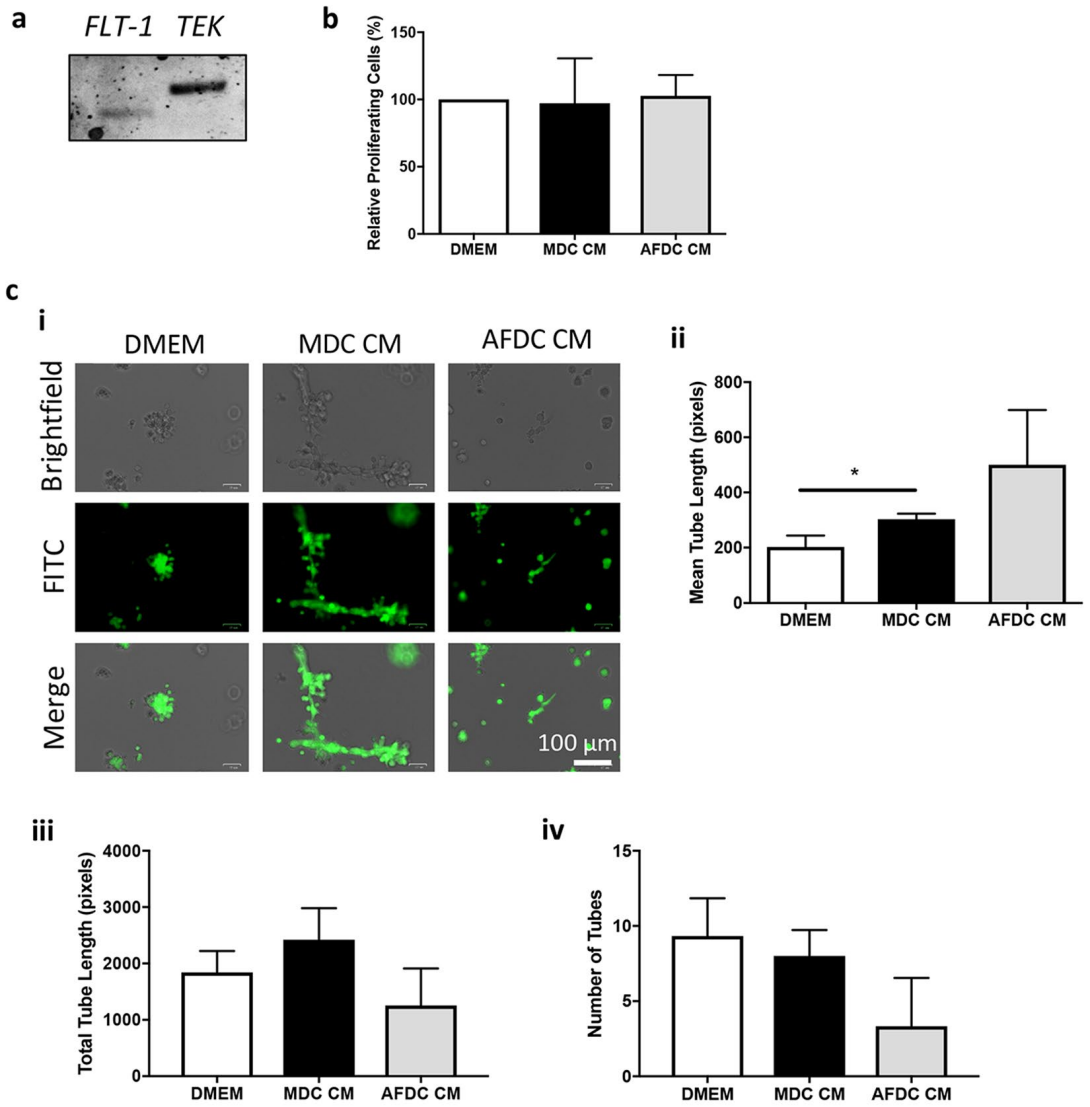
MDC CM stimulates endothelial tube-like formation *in vitro*. (**a**) Reverse transcription-polymerase chain reaction (RT-PCR) was used to evaluate the expression of the *FLT-1* (Angiopoietin-1 receptor) and *TEK* (Vascular Endothelial Growth Factor a receptor) in BLMVEC. RT-PCR products were run on a 1% agarose gel. (**b**) A bromodeoxyuridine (BrdU) proliferation assay was performed to evaluate the proliferation activity of BLMVEC after incubation with MDC or AFDC CM. BrdU incorporation was measured by determining the optical density at 450 nm on a Multiskan EX microplate reader using Ascent software (ThermoFisher Scientific). (**c**) BLMVEC were seeded on an extracellular matrix gel in the presence or absence of MDC or AFDC CM to evaluate the tube-like formation capacity *in vitro*. After 18 h of culture, BLMVEC were stained with 10 μl of 10X cell-based calcein. JNJ inhibitor was used as a negative control. Fluorescent and brightfield photographs were taken using a ZOE Fluorescent Cell Imager (BioRad). Representative images are shown (i). Images were quantified by Wim Tube Solutions for mean tube length (ii), total tube length (iii) and the number of tubes (iv). *p < 0.05.

Because we identified VEGFa and Ang-1 in the CM of MDC and AFDC, we evaluated the effects of the secretome on blood vessel formation. New blood vessels allow for a better supply of oxygen and nutrients to the damaged tissue, and as such, angiogenesis is an important aspect of the tissue repair process after damage^30^. First, we confirmed the presence of Fms related tyrosine kinase 1 (*FLT1*) and TEK receptor tyrosine kinase (*TEK*), the receptors for VEGFa and Ang-1, respectively, in bovine lung microvessel endothelial cells (BLMVEC) (Fig. 4a). Next, we used a bromodeoxyuridine (BrdU) proliferation assay to determine the effects of MDC and AFDC CM on BLMVEC proliferation. No difference was observed in proliferation between BLMVEC cultured in the presence or absence of CM (Fig. 4b). In contrast, when a luciferase-based assay was used to evaluate endothelial tube formation in the presence of MDC CM, AFDC CM, and non-conditioned Dulbecco’s Modified Eagle’s medium (DMEM), there was an increase in mean tube length in endothelial cells cultured in MDC CM compared to endothelial cells cultured in DMEM. A numerical increase was seen for cells cultured in AFDC CM, but statistical differences were not observed. (Fig. 4ci & ii). Total tube length as well as the number of tubes, were not altered in the presence of CM (Fig. 4ciii & iv).

### MDC CM increases bovine epithelial cell migration

**Figure 5:**
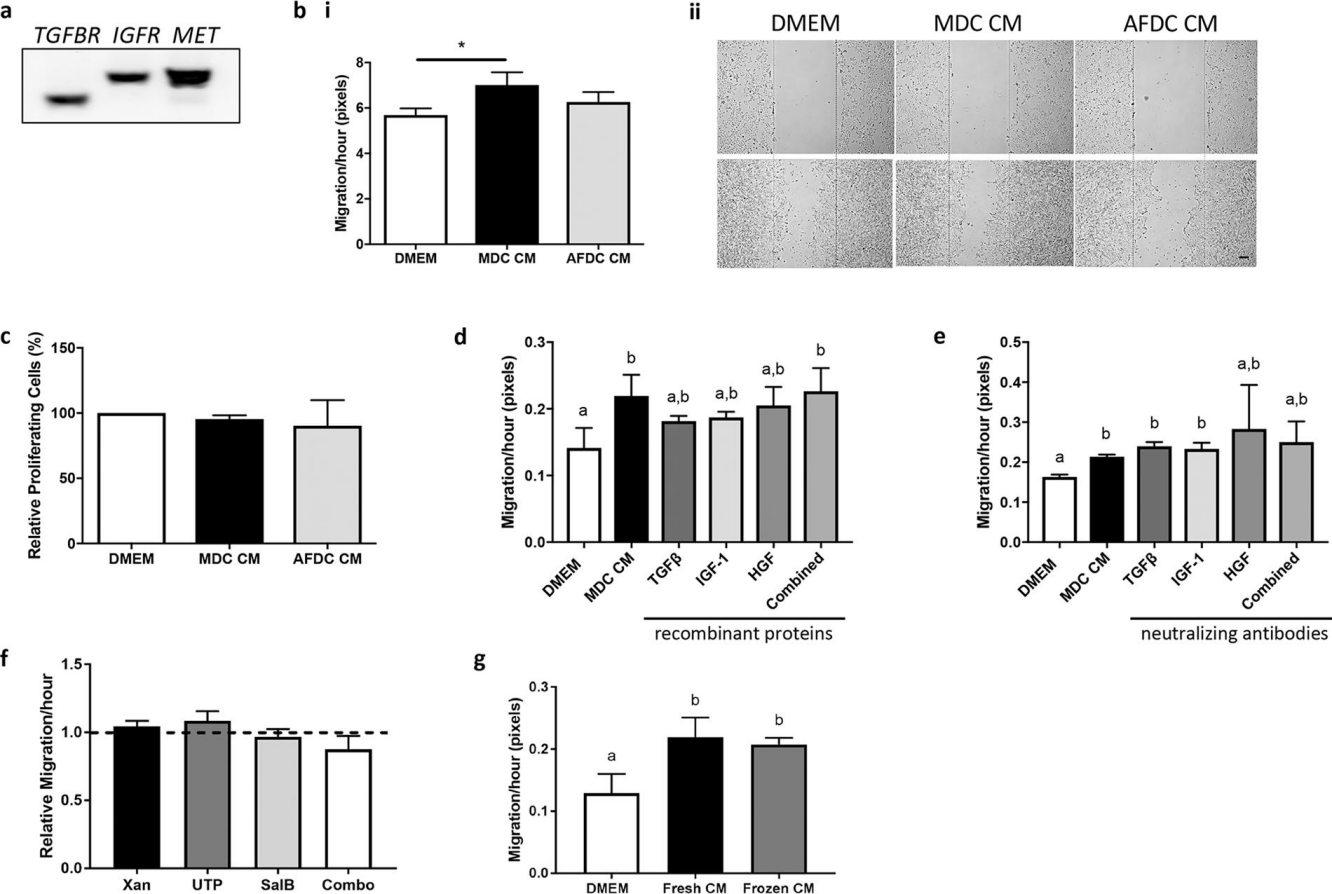
MDC CM stimulates epithelial cell migration but not proliferation. (**a**) RT-PCR was used to evaluate whether MDBK cells express receptors *TGFBR* (transforming growth factor beta receptor), *IGFR* (insulin-like growth factor receptor), and *MET* (hepatocyte growth factor receptor) for the migratory factors detected in the MDC and AFDC CM. RT-PCR products were run on a 1% agarose gel. (**b**) A scratch was made through a confluent layer of MDBK cells that were cultured with or without MDC or AFDC CM. Quantification of the rate of migration of MDBK cells incubated with MDC or AFDC CM using ImageJ software (i). Representative images of migration assays (ii). (**c**) A BrdU proliferation assay was performed to evaluate the proliferation activity of MDBK cells after incubation with MDC or AFDC CM. (**d**) Quantification of the rate of migration of MDBK cells cultured with or without MDC CM in the presence of TGFβ, IGF-1, HGF, or all (combined) recombinant proteins. (**e**) Quantification of the rate of migration of MDBK cells cultured with or without MDC CM in the presence of neutralizing antibodies for TGFβ, IGF-1, HGF, or all (combined). (**f**) Quantification of the rate of migration of MDBK cells cultured with CM from MDC treated with uridine triphosphate (UTP), salvionolic acid B (SalB), xanthosine (Xan), or all three (combo). Measurements are relative to untreated MDC CM indicated by the dotted line. (**g**) Quantification of the rate of migration of MDBK cells cultured with CM from MDC collected fresh (fresh CM) or frozen at −80 °C for one week and thawed (frozen CM). *p < 0.05. Different letters indicate statistically significant (*p* < 0.05*)* differences. Bar = 100 µm.

The final functional experiments performed assessed epithelial cell migration, as proteins with a role in cell migration were described in the mass spectrometry analysis (Table 1) and confirmed with ELISA (Table 2). As with proteins involved in angiogenesis, we first confirmed the presence of receptors on MDBK cells for the 3 proteins identified by ELISA, namely TGFβ receptor (*TGFBR*) for TGFβ, IGF-1 receptor (*IGFR*) for IGF-1, and MET proto-oncogene receptor tyrosine kinase (*MET*) for HGF using RT-PCR (Fig. 5a). *In vitro* scratch assays were used to evaluate the potential of MDC and AFDC CM to promote epithelial cell migration, and we found that MDBK cells migrated faster in the presence of MDC, but not AFDC, CM when compared to epithelial cells in DMEM (Fig. 5bi & ii). This effect was independent of cell proliferation, as we did not find an increase in the proliferation of epithelial cells when cultured in CM from either MDC or AFDC (Fig. 5c). To follow up on the increased rate of migration observed with the MDC CM, we repeated the scratch assays in the presence of commercially available recombinants of TGFβ, IGF-1, and HGF, alone or in combination. Given the statistical differences between each individual recombinant protein and the DMEM control, as well as between the combination of proteins and the DMEM control, it is likely that these proteins are contributing to the differences in migration rate that we observe when cells are treated with MDC CM (Fig. 5d). To further evaluate this, we treated the MDC CM with neutralizing antibodies for each protein, alone or in combination, and repeated the scratch assay. We observed a reduced migration in the presence of antibodies against HGF alone and the combination of all 3 antibodies, although this did not reach significance (Fig. 5e). Collectively, these results indicate that HGF, alone or in combination with TGFβ and IGF-1, at least in part contributes to cell migration.

Changes in the physiological environment of cells are known to alter the secretome^31^. For this reason, we decided to explore whether pretreating bovine MDC with stimulating agents would enhance the observed positive effect of their secretome on migration. To this end, we pre-stimulated MDC with xanthosine (Xan), Uridine triphosphatase (UTP), or Salvionolic acid B (SalB), alone or in combination, and collected the CM from these stimulated MDC for use in scratch assays. Xanthosine is a nucleoside analogue previously used to enhance bovine mammary stem/progenitor cells *in vitro* and *in vivo*^32,33^. UTP is also a natural nucleoside analogue, known to stimulate hematopoietic stem cells, and SalB is an antioxidant known to stimulate adult neural stem/progenitor cells^34,35^. Unfortunately, none of the MDC CM from individual pretreatments nor the combination of treatments, resulted in an increased migration rate of bovine epithelial cells when compared to the migration rate observed with unstimulated MDC CM (Fig. 5f).

While the culture of MDC is not technically challenging, the generation of CM does take several days. Storing CM long term for off-the-shelf use would be advantageous for treatment. As the effects of MDC CM were most pronounced on MDBK migration, we used this assay to test the efficacy of MDC CM after freezing at −80 °C for one week. We found that we can freeze the MDC CM without loss of activity on cell migration (Fig. 5g), which has important consequences for future clinical use in mastitis management.

## Discussion

This study characterized the secretome of bovine primary adherent fraction-derived cells (AFDC) and mammosphere-derived cells (MDC), and subsequently demonstrated the functionality of secreted factors involved in bacterial inhibition, angiogenesis and epithelial cell migration. As such, this study provides *in vitro* evidence of the therapeutic potential of primary bovine mammary cell conditioned medium (CM) in mastitis management.

Liquid chromatography-mass spectrometry identified several classes of antimicrobial peptides (AMP) secreted by MDC and AFDC. This is in line with previous studies from our lab and others, where the secretome of mesenchymal stem cells (MSC), a type of adult stem cell, was found to contain AMP and was able to inhibit the growth of bacteria^15,16,36^. In contrast to MSC CM, however, MDC CM had only a weak direct effect on bacterial growth and AFDC CM had no effect, at least for *Klebsiella (K*.*) pneumoniae*. Because the bacterial toxin lipopolysaccharide (LPS) produced by this bacterium is primarily responsible for the severe inflammation and sequelae of clinical signs and marked decreases in milk production^8,9^, we evaluated whether the secretome of MDC and AFDC could protect against LPS-induced epithelial damage. Our results showed that the CM from MDC and AFDC could protect bovine epithelial cells from LPS-induced damage. As previous studies have shown that the human cathelicidin cleavage product LL-37 can interfere directly with LPS^37^, and we did detect bovine cathelicidin in the secretome, we hypothesize that this AMP and/or its cleavage product myeloid antimicrobial peptide-27 (BMAP-27), the bovine homologue of human LL-37, is responsible for the observed protection against LPS-induced damage.

Besides AMPs, we also identified other secreted factors, more specifically those involved in angiogenesis and cell migration. For the latter, we decided to follow up on transforming growth factor beta (TGFβ), insulin-like growth factor 1 (IGF-1), and hepatocyte growth factor (HGF), based on their effects on epithelial cell survival and/or mitogenic action on epithelial cells^38–41^. Specifically, we treated epithelial cells with recombinant proteins of TGFβ, IGF-1, and HGF, and found a stimulating effect on migration when compared to epithelial cells cultured in complete MDC CM. However, the level of stimulation could only match the level of stimulation with the CM when all three recombinants were given simultaneously, highlighting one of the advantages of using whole CM over a treatment strategy using individual proteins. Additionally, inhibiting the activity of these proteins in MDC CM by pre-incubating the CM with neutralizing antibodies against TGFβ, IGF-1, and HGF, alone or in combination, only modestly reduced migration, indicating that other proteins contribute to this effect. The presence of various bioactive factors in the secretome with various effects on host defense and immunity, angiogenesis, and/or cell migration, further support the use of CM in its entirety as these factors act in concert to promote regeneration.

The natural nucleoside analogue xanthosine (Xan) has been shown to increase stem cell proliferation *in vitro* and to increase the number of stem cells in the mammary gland of heifers *in vivo*^32,33^. However, no information is available on the potential effect of Xan, and other nucleoside analogues such as uridine triphosphate (UTP) or the antioxidant salvionolic acid B (SalB), all used previously to stimulate stem cells^34,35^, on the composition of bioactive factors in the stem cell secretome. To test this, we pre-treated bovine MDC with these compounds, using similar concentrations as reported in the literature, and used their CM in *in vitro* scratch assays. When compared to the CM collected from MDC that were not pre-treated, no significant differences in migration rates were observed. Several reasons could explain the lack of increased migratory activity of the CM from these pre-treated cells. For example, the effect of the stimulant may have been short and transient and, therefore, no longer present 24 hours after changing the media, when CM was collected for experiments. Another explanation could simply be that an increase in cell proliferation does not correlate with an increased secretory capacity of factors involved in cell migration and/or was insufficient to be translated into a functional effect. Transcriptional analyses of MDC stimulated with these compounds would provide useful information about which genes are upregulated. Genes involved in migration and other functions could subsequently be quantified on a protein level in the MDC secretome and studied in more detail. In contrast, we were able to demonstrate that freezing and thawing of the MDC CM did not adversely affect the migration stimulation when compared to fresh CM. Based on these results, one could anticipate that the CM of MDC can be formulated as a readily available, shelf-stable, biological product to be used in intramammary preparation. The use of CM, in general, negates the risk of rejection when using the cells themselves as a therapeutic^42^. Still, future research is needed to confirm the beneficial effects of the MDC secretome *in vivo*, particularly with regards to the effects of competing *in vivo* factors, such as immune cells and the presence of milk proteins, on the efficacy of the CM. Moreover, the duration of efficacy of the CM will be important to evaluate, as the half-live of many growth factors, such as those found in this study, tends to be short^43^.

Taken together, our study provides the rationale for the potential use of the MDC secretome as an adjunct therapy in mastitis management. The functionality of secreted proteins, combined with the ease and low cost of isolating and culturing bovine MDC, makes the secretome an ideal biological source for naturally-occurring peptides as well as other bioactive factors.

## Materials and Methods

### Cells

Bovine primary mammary adherent fraction-derived cells (AFDC) and mammosphere-derived cells (MDC) were isolated as follows. Mammary gland tissues from clinically healthy, non-lactating heifers were collected after euthanasia by excising 2 parts of 5 cm^2^ of tissue next to the median line of the two mammary gland compartments. Samples were dissociated mechanically with a sterile scalpel, followed by enzymatic digestion with 0.1% collagenase III (Worthington Biochemical Corporation, Lakewood, NJ) at 37 °C for 60 min. The cell suspension was subsequently filtered through sterile 100 and 40 μm filters to obtain a single cell suspension, and centrifuged at 400 × g for 10 min at room temperature (RT). Cells were resuspended in phosphate buffered saline (PBS) with 1% penicillin-streptomycin (P/S), centrifuged at 260 × g for 10 min, and resuspended in MDC medium, consisting of Dulbecco’s modified Eagle’s medium (DMEM)/F12 (50/50) supplemented with 10% fetal bovine serum (FBS), 2% B27 (all from Invitrogen, Carlsbad, CA), 1% penicillin/streptomycin (P/S), 10 ng/mL basic-fibroblast growth factor (BioVision, Milpitas, CA), and 10 ng/mL epidermal growth factor (Sigma-Aldrich, St. Louis, MO). Approximately 1 × 10^6^ cells were seeded on a 6-well tissue culture dish for 1 h and this was repeated once more. The adherent cells were further propagated as AFDC. The non-adherent cells were collected and seeded at approximately 20,000 cells/cm^2^ on 6-well ultralow attachment plates (Elscolab, Corning, NY). MDC medium was refreshed twice a week by means of centrifugation of the mammospheres at 300 × g for 7 min. For further experiments, mammospheres were seeded on adhesive tissue culture dishes in MDC medium unless where indicated otherwise. The bovine cell lines Madin-Darby bovine kidney epithelial (MDBK) and bovine lung microvessel endothelial cells (BLMVEC) were cultured in DMEM supplemented with 10% FBS (Atlanta Biologicals, Lawrenceville, GA) and 1% P/S. All cell lines were maintained at 37 °C in a humidified environment with 5% CO_2_.

### Immunostaining

Fixed cultures were incubated overnight at 4 °C with primary antibodies diluted in PBS, then washed and incubated for 1 hour at room temperature with a fluorescently labeled secondary antibody. Primary antibodies used included mouse monoclonal RV202 anti-vimentin, mouse monoclonal LL002 anti-CK14 (Abcam, Cambridge, MA), mouse monoclonal 1A4 anti-alpha smooth muscle actin (Dako, Santa Clara, CA), mouse monoclonal TE111.5D11 anti-estrogen receptor alpha, mouse monoclonal C-04 anti-CK18 (ThermoFisher Scientific, Waltham, MA), and rabbit polyclonal anti-β-lactoglobulin (diluted 1:100; Abcam). Secondary antibodies used included Alexa Fluor 488-Goat Anti-Mouse IgG or Alexa Fluor 488-Goat Anti-Rabbit IgG (both diluted 1:250, Jackson ImmunoResearch, West Grove, PA). DAPI was used to stain nuclei. Stained cultures on coverslips were mounted on glass slides and visualized using a confocal laser scanning microscope (Zeiss, Oberkochen, Germany). β-lactoglobulin-stained acini were visualized in a ZOE Fluorescent Cell Imager (BioRad, Hercules CA).

### Flow cytometric analyses

Cells were collected using accutase (Innovative Cell Technologies, San Diego, CA) and stained with mouse monoclonal TDM29 anti-CD29 (diluted 1:50; Sigma-Aldrich), rat monoclonal IM7 anti-CD44 (diluted 1:20; ThermoFisher Scientific), or rat monoclonal GoH3 anti-CD49f (diluted 1:10; BD Biosciences, San Jose, CA) antibodies diluted in PBS with 1% BSA, for 1 h. Staining with an isotype control antibody (Abcam), diluted 1:10 in PBS with 1% BSA, or no antibody stain were included as controls. Cells were washed three times in PBS and incubated with Alexa 488-conjugated secondary goat anti-rabbit and goat anti-mouse antibodies diluted 1:500 in PBS with 1% BSA, for 30 min. After washing, 50,000 cells were analyzed on a Gallios flow cytometer controlled by Kaluza for Gallios software (Beckman Coulter, USA). Data analysis was conducted using Kaluza Analysis software (Beckman Coulter).

### Acinar differentiation assays

Acinar differentiation assay followed the methods of Dontu *et al*. (2003), with slight modification^17^. Bovine MDC were seeded on wells pre-coated with Matrigel (Corning), at a density of 53 cells/cm^2^ (100 cells/well in a 24-well plate) and cultured in MDC medium. After 7 days, a layer of Matrigel was added in addition to fresh medium containing prolactin (Sigma-Aldrich) at 1 µg/mL. Seven days later, 3D acinar structures were fixed *in situ*, stained for β-lactoglobulin, and imaged with the ZOE Fluorescent Cell Imager (as described above).

### Generation of conditioned medium (CM) and Pretreatments

CM was collected from MDC and AFDC after 2 days of culture, when cells were 70% confluent. To this end, 1 × 10^6^ cells were seeded in a T75 flask in 10 mL of DMEM + 10% FBS + 1% P/S. After 24 h, cells were washed twice with PBS and medium was changed to DMEM without serum or antibiotics. Twenty-four hours later, the supernatant was collected and, after centrifugation twice for 10 min at 400 × g, used for additional experimentation.

For the pre-treatment experiments, MDC were seeded in MDC medium supplemented with 200 µM xanthosine (Xan), 10 µM uridine triphosphatase (UTP), or 50 µM salvionolic acid B (Sal B), all from Sigma-Aldrich. After 24 h of culturing, the cells were washed twice with PBS and medium was changed to DMEM without serum or antibiotics. CM was collected 24 h later as described.

### Mass Spectrometry (MS)

In-solution digestion was performed following a protocol by Zhang *et al*. (2003) with slight modification^44^. The protein pellet from 10 mL of medium culture was dissolved in 300 µL denaturing solution containing 6.0 M guanidine-hydrochloride, and 100 mM PBS pH 7.0, sonicated for 5 min, centrifuged, and supernatant was collected. The denatured sample was reduced with 10 mM dithiothreitol and alkylated with 55 mM iodoacetamide for 1 h at RT. Excess iodoacetamide was quenched with 30 mM dithiothreitol for 30 min at RT. The samples were diluted to a final volume of 3,775 µL with 50 mM Tris hydrochloride pH 8.0 to reduce the guanidine-hydrochloride concentration to less than 1 M. The samples were digested by adding 25 µg trypsin (Promega, Madison, WI) and incubated at 37 °C for 16 h. Digestions were stopped by the addition of 25 µL trifluoroacetic acid. The digests were further desalted by solid phase extraction using Sep-Pack Cartridge (Waters Corporation, Milford, MA) and the eluted peptides were evaporated to dryness by a Speedvac SC110 (Thermo Savant, Milford, MA). The data-dependent acquisition raw files for collision-induced dissociation tandem mass spectrometry only were subjected to database searches using Proteome Discoverer 2.2 software (ThermoFisher Scientific) with the Sequest HT algorithm.

### Western Blot analyses

CM was collected, as described above, and incubated with a 1X general protease inhibitor. Protein concentration was determined with a bicinchoninic acid protein assay (ThermoFisher Scientific) prior to gel loading to ensure loading of an equal amount of protein (40 μg). 6X reducing sample buffer was added to yield a final concentration of 1X and lysates were boiled for 10 min at 95 °C. Samples were subjected to sodium dodecyl sulfate polyacrylamide gel electrophoresis on a 12% gel and transferred to immobilin polyvinylidine difluoride membranes (Millipore, Billerica, MA) using a transblot turbo system (Biorad). Membranes were blocked in 5% BSA diluted in Tris buffered saline (TBS) and incubated with lactoferrin (LTF) antibodies diluted 1:50 or bovine cathelicidin (Cath) antibodies diluted 1:1000 in TBS + 5% BSA for 1 h at RT on a rotating platform. Blots were washed for 50 min (10 × 5 min) with TBS-Tween, then incubated with a 1:20,000 dilution of HRP-conjugated goat anti-rabbit or HRP-conjugated goat anti-mouse antibodies for 1 h at RT. All blots were washed for 50 min (10 × 5 min) with TBS-Tween and then visualized by chemiluminescence using Clarity Western ECL (BioRad). Gels were imaged on a BioRad ChemiDoc MP system (BioRad) and band intensities were determined using Image Lab software.

### Enzyme-Linked Immunosorbent Assays (ELISA)

The concentration of VEGF, ANG-1, TGFβ, IGF-1, and HGF in CM was determined in duplicate wells by sandwich ELISA, following the manufacturer’s guidelines for each kit (VEGF: Sigma-Aldrich; ANG-1, TGFβ, IGF-1, HGF: LifeSpan BioSciences, Inc., WA).

### Gene expression analysis

Cells were seeded at a density of 2 × 10^5^ in T25 tissue culture flasks. After 48 h, mRNA was extracted from the cells using an RNEasy Plus Kit (QIAGEN, Valencia CA) and cDNA was synthesized using M-MLV Reverse Transcriptase (USB, Cleveland, OH), both according to manufacturer’s protocols. The amplification reactions were performed using Invitrogen reagents (Life Technologies, Grand Island, NY) and an Eppendorf Mastercycler. Following standard gel electrophoresis, products were visualized using a ChemiDoc MP Imaging System (Bio-Rad, Hercules, CA). Primers to amplify *FLT-1*, *TEK*, *TGFβR*, *MET* and *IGF-1R* were designed using Primer3 software, based on bovine sequences found in the National Center of Biotechnology Information (NCBI) GenBank. Primer sequences are listed in Table 3.

**Table 3:** Primers used for gene expression analyses.

Gene Product	Abbreviation	Forward Sequence (5′ → 3′)	Reverse Sequence (5′ → 3′)
Fms related tyrosine kinase 1	*FLT1*	AACACAAGAGTTGAGATGACCTG	TGACAATCAGAGTGACAGTGAAG
TEK receptor tyrosine kinase	*TEK*	CTGTTAATCACTATGAGGCTTGG	TAAAAGTCATCTTCTGAGCTTGG
Transforming growth factor beta receptor	*TGFBR*	GACCAGTCTGCTTTGTCTGTATC	GTCTGATAAATCTCTGCTTCACG
Insulin-like growth factor receptor	*IGFR*	TTACTCTGTACCGAATCGACATC	TTATAACCAAGCCTCCCACTATC
MET proto-oncogene, receptor tyrosine kinase	*MET*	ATGGTAATAAATGTGCATGAAGC	ACAATCAATCCTGTGAAATTCTG

### Bacteria

All bacteria were acquired from the Quality Milk Production Services (QMPS) at Cornell University, Ithaca, NY and were kept as frozen stocks in glycerol. One isolate of the known mastitis-causing field strain of *Klebsiella pneumoniae* was acquired from a NYS dairy farm. Bacteria were streaked on trypticase soy agar containing 5% sheep blood and 1% esculin (PML Microbiologicals, Canada) and incubated for 24 h at 37 °C for the isolation of single colonies prior to the start of experiments. A selected colony was inoculated and grown for 16–18 h overnight in 5 mL Luria-Bertani (LB) broth (ThermoFisher Scientific) with constant shaking at 37 °C. Antibacterial activity was measured by a microdilution susceptibility test using 96 well plates. A 50% dilution of conditioned media from 3 AFDC cultures and 3 MDC cultures was aliquoted into wells (90 uL) in triplicate. Subsequently, a bacterial suspension comprised of 10 µL of LB broth containing 100 CFUs was added to the CM. The plates were incubated for 8 h with constant shaking at 37 °C. Spectrophotometer readings were taken as optical densities (OD_600_). Values were expressed as percent O.D. relative to a control of cell-free DMEM and bacteria. The three wells corresponding to each dilution were combined, a 10 µL sample was removed from the composite, diluted serially, plated on LB agar, and incubated overnight at 37 °C for quantification of colony growth as colony forming units (CFU).

### Viability assays

After 48 h of treatment with either liposaccharide (LPS) or alpha toxin (AT) in the presence of MDC CM, AFDC CM, or DMSO (control), a 3-(4,5-dimethylthiazol-2-yl)-2,5-diphenyltetrazolium bromide (MTT) *in vitro* toxicology assay (Sigma-Aldrich) and lactate dehydrogenase (LDH) assay (Sigma-Aldrich) were carried out as per manufacturer’s instructions and absorbance was measured at 570 nm or 490 nm respectively on a Multiskan EX plate reader (ThermoFisher Scientific). Values were expressed relative to untreated wells.

### Bromodeoxyuridine proliferation assay

The effect of CM on the proliferation of MDBK and BLMVEC was evaluated using a bromodeoxyuridine (BrdU) proliferation assay kit (Abcam). Cells were seeded on a 96 well plate at a density of 4 × 10^4^ cells per well and incubated with unconditioned medium (control), MDC CM, or AFDC CM for 48 h. The BrdU proliferation assay kit was used according to the manufacturer’s instructions and the resulting absorbance was read at 450 nm using a Multiskan EX microplate reader and Ascent software (ThermoFisher Scientific). Empty wells and wells without BrdU were included as controls.

### Angiogenesis Assay

Endothelial tube-like formation assays were performed using an *in vitro* angiogenesis assay kit, according to the manufacturer’s instructions (Cayman Chemicals, Ann Arbor, MI). BLMVEC were seeded in triplicate onto an extracellular matrix gel (1 × 10^4^ cells per well) and incubated in 100 µl of either MDC or AFDC CM or DMEM to establish the baseline BLMVEC growth or 1 μM JNJ-10198490 was used as a negative control, according to the manufacturer’s instructions. After 18 h of culture, the BLMVEC were stained with 10 μl of 10X cell-based calcein (Cayman Chemicals) and the presence of tree-like tubular networks was examined using a ZOE Fluorescent Cell Imager (BioRad).

### *In vitro* scratch assay

Scratch assays were carried out as previously described^45^. MDBK cells were seeded at 8,000 cells/cm^2^. After 24 h, a line in the monolayer was performed with a sterile p200 pipette tip and medium was changed to DMEM, MDC CM, or AFDC CM. Zero, 24, and 48 h post scratching images were captured using a Nikon Diaphot-TMD inverted light microscope with an attached Cohu CCD camera (Nikon, Melville, NY). Image J software (http://rsbweb.nih.gove/ij/) was used to measure the distance of the scratch.

### Recombinant Proteins and Neutralizing Antibodies

The recombinant proteins used in the present study include bovine TGFβ (1916 pg/ml), (LifeSpan Biosciences, Inc., WA), bovine IGF-1 (82 pg/ml) and human HGF (57 pg/ml; ThermoFisher Scientific). The neutralizing antibodies used in the present study include TGFβ (MM0446-6G14), IGF-1 (SPM406), and HGF (MM0326-5D20) from Novus Biologicals (Littleton, CO) at a concentration of 50 times the amount reported by ELISA.

### Statistical analyses

The Student’s *t* test for unpaired data was used to evaluate differences between the means when comparing MDC to AFDC (cells or CM) for the following analyses: percentage of expression in flow cytometry, band intensity in Western blot analyses, protein quantifications in ELISAs, viability assays analyses, BrdU analyses, and migration assays. One-way ANOVA, followed by the Tukey’s multiple comparison test, was used to assess differences in migration rates between recombinant proteins, neutralizing antibodies, stimulated CM, and frozen CM. Where not specified, a value of *P* < 0.05 was considered statistically significant. GraphPad software was used for analysis (GraphPad Software, La Jolla, CA, USA). Data given are the mean of three replicates and the bars show standard deviation.

## Electronic supplementary material

Supplemental Figure 1
