# Supplementary material for: The secretome from bovine mammosphere-derived cells (MDC) promotes angiogenesis, epithelial cell migration, and contains factors associated with defense and immunity
Source: Sci Rep. 2018 Mar 29;8:5378. doi: 10.1038/s41598-018-23770-z (PMC5876384; doi:10.1038/s41598-018-23770-z)
Supplement: Supplementary file 1 — Supplemental Figure 1 [file 41598_2018_23770_MOESM1_ESM.pdf]

SUPPLEMENTARY INFORMATION

The secretome from bovine mammosphere-derived cells (MDC) promotes angiogenesis, epithelial cell migration, and contains factors associated with defense and immunity.

Melissa M. Ledet, Amy K. Vasquez, Gat Rauner, Allison A. Bichoupan, Paolo Moroni, Daryl V. Nydam, and Gerlinde R. Van de Walle

Supplemental Figure 1. Schematic figure depicting how two separate primary cell populations (AFDC and MDC) were isolated from fresh bovine mammary tissue through differential surface adherence and propagation in suspension culture. The quick-adhering cells (AFDC) were separated from the slow-adhering cells, which were then cultured in suspension culture. Suspended cells then formed floating colonies (mammospheres) when cultured on low-attachment plates, indicative of their ability to survive in non-adherent conditions. Mammospheres were dissociated to single cells that were then cultured as a monolayer in adherent culture (MDC). Bar = 100μm.

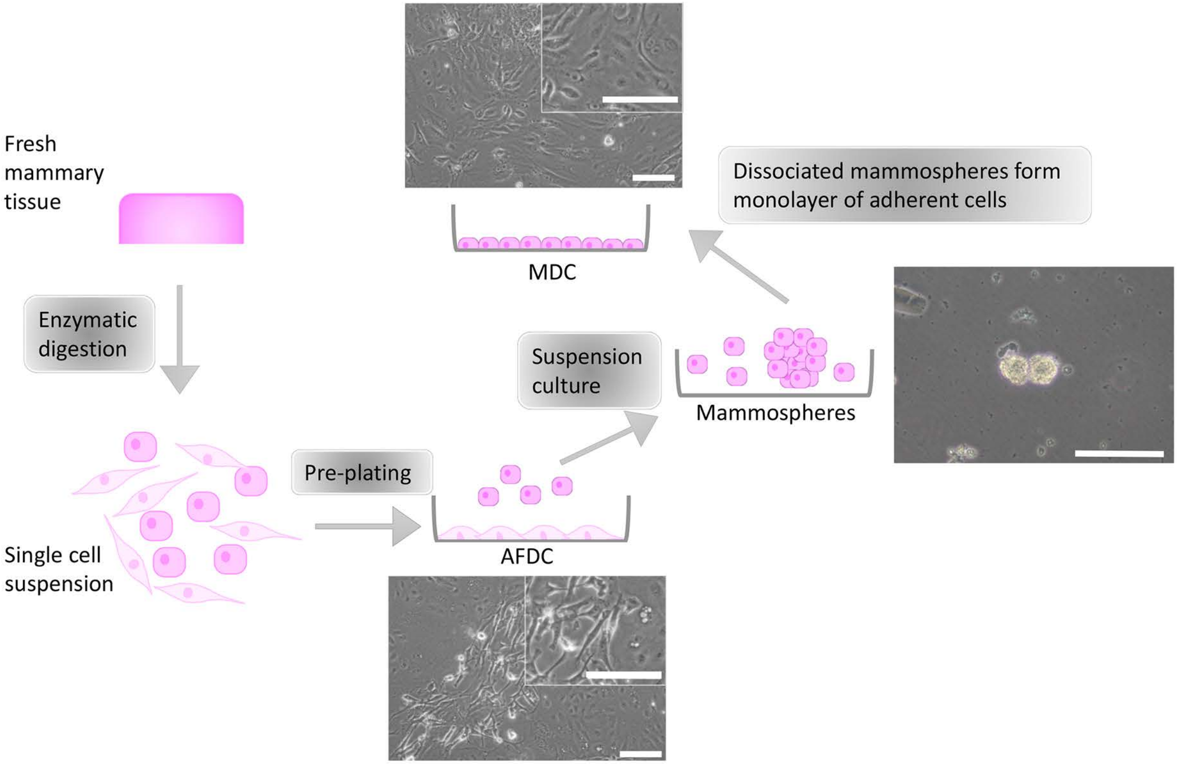
Fresh mammary tissue
Enzymatic digestion
Single cell suspension
Pre-plating
AFDC
Suspension culture
Mammospheres
Dissociated mammospheres form monolayer of adherent cells
MDC
